# Severity and geographical disparities of post-COVID-19 symptoms among the Vietnamese general population: a national evaluation

**DOI:** 10.1038/s41598-023-30790-x

**Published:** 2023-03-17

**Authors:** Bach Tran, Minh Ngoc Le Vu, Huong Thi Le, Tu Huu Nguyen, Laurent Boyer, Guillaume Fond, Pascal Auquier, Carl A. Latkin, Roger C. M. Ho, Cyrus S. H. Ho, Melvyn W. B. Zhang

**Affiliations:** 1 EA 3279, CEReSS, Research Centre On Health Services and Quality of Life, Aix Marseille University, 27, Boulevard Jean-Moulin, 13385 Marseille Cedex 05, France; 2 Institute for Preventive Medicine and Public Health, Hanoi Medical University, Hanoi, 100000 Vietnam; 3 Institute of Health Economics and Technology (iHEAT), Hanoi, 100000 Vietnam; 4 Vietnam Young Physicians Association, Hanoi, 100000 Vietnam; 5 Bloomberg School of Public Health, Johns Hopkins University, Baltimore, MD 21205 USA; 6 Department of Psychological Medicine, Yong Loo Lin School of Medicine, National University of Singapore, Singapore, 119228 Singapore; 7 Institute for Health Innovation and Technology (iHealthtech), National University of Singapore, Singapore, 119077 Singapore; 8 Lee Kong Chian School of Medicine, Nanyang Technological University, Singapore, 639815 Singapore

**Keywords:** Diseases, Health care, Medical research, Signs and symptoms

## Abstract

Post-COVID-19 symptoms have become a significant global health concern. This study focused on assessing the prevalence, severity, and care preference of post-COVID-19 symptoms, as well as identifying determinants to inform evidence-based policy on post-COVID-19 in Vietnam. A national cross-sectional study was conducted in May 2022 among 12,361 recovered COVID-19 patients, providing the largest dataset on health status after COVID-19 in Vietnam. The study utilized ordered logistic, Poisson regression, Multilevel linear random-effects models, and Multilevel random effects ordered logistic model to identify factors associated with various aspects of post-COVID-19 conditions. Results showed that the average number of post-COVID-19 symptoms was approximately 3, with fatigue and headache being the most common symptoms. The number of post-COVID-19 symptoms varied by province, decreased with age, and was significantly correlated with the duration of infection. Age, infection period, underlying conditions, telehealth utilization, and geographical location were identified as significant determinants of post-COVID-19 symptoms. The study concluded that improving resource allocation and health-seeking behavior in underserved areas could help address differences in health outcomes and improve post-COVID-19 control in Vietnam.

## Introduction

Following nearly three years of unprecedented global spread, the COVID-19 pandemic has now entered its post-peak period. At the start of June, global data reported 225,120 new cases daily and a 99% recovery rate, compared to the peak in November 2021, which saw 514,914 new cases daily and a 90.14% recovery rate^1,2^. In Vietnam, due to rapid response strategies and a comprehensive national vaccination program, the country has been able to considerably control the course of the pandemic^3,4^. As of June 2022, Vietnam recorded an average of only 910 new cases daily, and there were four deaths in six consecutive weeks^5^. However, the slowing of the spread does not signify the end of the pandemic but rather the beginning of a new era in the fight against COVID-19. The aftermath of the virus includes economic losses, environmental degradation, increased social inequality, disruption to lifestyles, and, most significantly, long-term or even permanent health consequences^6–8^.

The World Health Organization (WHO) has defined post-COVID-19 condition as a collection of long-term symptoms that can persist after being infected by COVID-19, either from the initial illness or after recovery. These symptoms may be less severe than those experienced during the initial infection, but they can affect multiple organs simultaneously, including the respiratory, cardiovascular, gastrointestinal, and neurological systems^9–11^. Research suggests that approximately 68% of COVID-19 survivors experience post-COVID-19 symptoms, and the duration of these symptoms can range from days to several months or even years^12^. However, investigating post-COVID-19 symptoms is challenging because of the lack of a standardized protocol, confirmed risk factors, and endpoints, which limits the body of evidence on this condition^14,15^. Despite several proposed guidelines for the diagnosis and management of post-COVID-19 symptoms, interventions remain in the early stages, with emergency recommendations being more common than evidence-based approaches. Moreover, high operational costs and exhausted workforces after a prolonged period of overwork make these interventions hardly feasible in the long term^16–19^. Existing frameworks for post-COVID-19 intervention also have limitations, with a lack of contextual domains for resource-scarce regions in low- and middle-income countries being a common barrier.

The Ministry of Health in Vietnam has identified more than 200 post-COVID-19 symptoms that have an extended impact on national health, safety, and socioeconomic growth^20^. Nevertheless, there is still a dearth of comprehensive data on the prevalence and impact of post-COVID-19 symptoms on the Vietnamese population, which impedes the creation of a national strategy and exacerbates the health disparity between regions in Vietnam. Our study represents one of the largest and earliest efforts to investigate the residual effects of COVID-19 on the Vietnamese population. Our objective was to determine the frequency and severity of post-COVID-19 symptoms in Vietnam, and to analyze the patterns and factors that contribute to a post-COVID-19 symptom timeline. Our findings will not only add to the growing corpus of research on post-COVID-19 symptoms but also facilitate the development of evidence-based interventions in Vietnam in the near future.

## Results

Table 1 presented the individual characteristics of the participants. The majority of respondents were male (65.1%), and the median age was 18 (IQR = 17–27). More than one-third of respondents resided in Northern Midlands and Mountains (34.7%), and 22.1% resided in the Mekong Delta regions. Only 4.9% of respondents were smokers, while 19.7% consumed alcohol. A large proportion of respondents (70.8%) exercised after recovery, and 63.1% had a normal BMI Index. Only a minor proportion (3.1%) had comorbidities.

**Table 1:** Individual characteristics of participants.

Characteristics	n	%
Gender		
Male	8035	65.1
Female	4305	34.9
Provinces		
Southeast region	549	4.8
Northern midlands and mountains	3985	34.7
Red river delta	1131	9.9
North central region	493	4.3
South central coast	1839	16.0
Central highlands	935	8.2
Mekong delta regions	2540	22.1
Smoking	594	4.9
Alcohol use	2415	19.7
Exercising after recovering from COVID-19	8693	70.8
BMI index		
Underweight	3382	28.2
Normal	7578	63.1
Overweight/Obese	1047	8.7
Comorbidities	378	3.1
Diabetes	28	0.2
Cardiovascular disease	208	1.6
Chronic lung disease	88	0.6
Neurological disease	59	0.4
Cancer	39	0.3
	**Median**	**IQR**
Age (range 16–35)	18	17–27

Table 2 showed that more than two-thirds of respondents were infected with COVID-19 in the recent 1 to 4 months. Participants infected with COVID-19 for less than 7 days had the highest proportion (49.8%), followed by 7 to 14 days (48.1%). In terms of the severity of COVID-19 at the onset, respondents who had mild symptoms had the highest percentage of 79.8%, and 10.8% of respondents were asymptomatic. The prevalence of cases in the region (per 100,000 population) and COVID-19 case fatality rate were calculated as 13,695.5 (IQR = 4437.2–17,936.5) and 0.08% (IQR = 0.01%–0.46%) in our sampled region. There were statistically significant differences between regions (*p* < 0.001).

**Table 2:** Characteristics of participants when infected with COVID-19 by province.

Characteristics	Southeast region	Northern midlands and mountains	Red river delta	North central region	South central coast	Central highlands	Mekong delta regions	Total	*p*-value
	n (%)	n (%)	n (%)	n (%)	n (%)	n (%)	n (%)	n (%)	
Time since COVID-19 onset									
1 month	78 (14.3)	704 (17.9)	129 (11.4)	98 (20)	210 (11.5)	189 (20.5)	314 (12.5)	1722 (15.1)	< 0.001
1–4 months	374 (68.4)	2773 (70.4)	870 (77.2)	351 (71.5)	1386 (75.6)	674 (72.9)	1590 (63.2)	8018 (70.5)	
4–6 months	59 (10.8)	159 (4)	75 (6.7)	20 (4.1)	124 (6.8)	34 (3.7)	394 (15.7)	865 (7.6)	
Above 6 months	36 (6.6)	305 (7.7)	53 (4.7)	22 (4.5)	113 (6.2)	27 (2.9)	218 (8.7)	774 (6.8)	
COVID-19 infection period									
Less than 7 days	279 (50.9)	2147 (54.3)	541 (48.1)	215 (43.8)	897 (48.9)	393 (42.6)	1209 (47.8)	5681 (49.8)	< 0.001
7–14 days	259 (47.3)	1736 (43.9)	552 (49.1)	269 (54.8)	897 (48.9)	511 (55.4)	1264 (50)	5488 (48.1)	
More than 14 days	10 (1.8)	70 (1.8)	32 (2.8)	7 (1.4)	41 (2.2)	19 (2.1)	54 (2.1)	233 (2)	
Severity of COVID-19 at the onset									
Asymptomatic	57 (10.4)	408 (10.2)	148 (13.1)	38 (7.7)	209 (11.4)	90 (9.6)	288 (11.3)	1238 (10.8)	< 0.001
Mild	456 (83.1)	3145 (78.9)	890 (78.7)	396 (80.3)	1491 (81.1)	741 (79.3)	2030 (79.9)	9149 (79.8)	
Moderate	30 (5.5)	403 (10.1)	85 (7.5)	52 (10.5)	129 (7)	99 (10.6)	207 (8.1)	1005 (8.8)	
Severe	6 (1.1)	29 (0.7)	8 (0.7)	7 (1.4)	10 (0.5)	5 (0.5)	15 (0.6)	80 (0.7)	
	**Median (IQR)**	**Median (IQR)**	**Median (IQR)**	**Median (IQR)**	**Median (IQR)**	**Median (IQR)**	**Median (IQR)**	**Median (IQR)**	**p-value**
Prevalence of case in region (per 100,000 population)	11,565.3 (11,501.5–11,565.3)	17,936.5 (17,936.5–17,936.5)	19,055.7 (16,708.6–19,055.7)	13,695.5 (3862.9–13,695.5)	4877.6 (3851.8–8315.2)	11,179.7 (4437.2–11,179.7)	2808.2 (2808.2–7527.9)	13,695.5 (4437.2–17,936.5)	< 0.001
COVID-19 case fatality rate (%)	0.67 (0.19–0.67)	0.01 (0.01–0.01)	0.08 (0.058–0.08)	0.06 (0.06–0.10)	0.31 (0.27–0.32)	0.06 (0.06–0.17)	1.84 (1.24–2.22)	0.08 (0.01–0.46)	< 0.001

**Figure 1:**
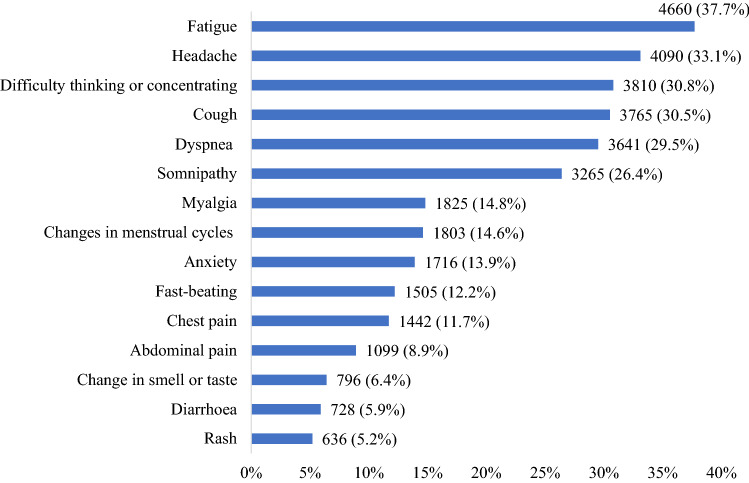
Prevalence of post-COVID-19 symptoms.

From Fig. 1, fatigue was the most common symptom of participants (37.7%), followed by headache (33.1%). Moreover, symptoms such as difficulty thinking or concentrating, cough, dyspnea, and somnipathy had a high percentage of 30.8%, 30.5%, 29.5%, and 26.4%, respectively.

Table 3 revealed that neurological symptoms were the most frequent symptoms of post-COVID-19 across all regions, followed by respiratory and heart symptoms. In general, approximately one-third (31.1%) of participants had four or more four symptoms, while in the red river delta region, the highest proportion (33.4%) of participants were asymptomatic. The mean number of neurological symptoms, digestive symptoms, respiratory and heart symptoms, and other symptoms were 1.43 (SD = 1.45), 0.21 (SD = 0.54), 0.84 (SD = 1.04), and 0.35 (SD = 0.62), respectively. The average of post-COVID-19 symptoms was 2.82 (SD = 2.80). Residents in Northern Midlands and Mountains had the highest number of both four types of symptoms (neurological, digestive, respiratory and heat, and others), there were statistically significant differences between regions (*p* < 0.001).

**Table 3:** Characteristics of post-COVID-19 symptoms by province.

Characteristics	Southeast region	Northern midlands and mountains	Red river delta	North central region	South central coast	Central highlands	Mekong delta regions	Total	*p*-value
	n (%)	n (%)	n (%)	n (%)	n (%)	n (%)	n (%)	n (%)	
Neurological symptoms	373 (67.9)	2673 (67.1)	568 (50.2)	356 (72.2)	1225 (66.6)	660 (70.6)	1579 (62.2)	7434 (64.8)	< 0.001
Digestive symptoms	64 (11.7)	800 (20.1)	125 (11.1)	74 (15.0)	265 (14.4)	162 (17.3)	316 (12.4)	1806 (15.7)	< 0 .001
Respiratory and heart symptoms	311 (56.6)	2107 (52.9)	483 (42.7)	246 (49.9)	899 (48.9)	482 (51.6)	1309 (51.5)	5837 (50.9)	< 0.001
Other symptoms	151 (27.5)	1250 (31.4)	248 (21.9)	136 (27.6)	486 (26.4)	280 (29.9)	632 (24.9)	3183 (27.7)	< 0.001
Number of post-COVID-19 symptoms									
Asymptomatic	89 (16.2)	800 (20.1)	378 (33.4)	69 (14.0)	337 (18.3)	157 (16.8)	537 (21.1)	2367 (20.6)	< 0.001
One symptom	125 (22.8)	748 (18.8)	230 (20.3)	116 (23.5)	430 (23.4)	185 (19.8)	549 (21.6)	2383 (20.8)	
Two symptoms	86 (15.7)	529 (13.3)	169 (14.9)	79 (16.0)	303 (16.5)	158 (16.9)	441 (17.4)	1765 (15.4)	
Three symptoms	84 (15.3)	471 (11.8)	121 (10.7)	74 (15.0)	215 (11.7)	135 (14.4)	284 (11.2)	1384 (12.1)	
Four or more than four symptoms	165 (30.1)	1437 (36.1)	233 (20.6)	155 (31.4)	554 (30.1)	300 (32.1)	729 (28.7)	3573 (31.1)	
	**Mean (SD)**	**Mean (SD)**	**Mean (SD)**	**Mean (SD)**	**Mean (SD)**	**Mean (SD)**	**Mean (SD)**	**Mean (SD)**	***p*-value**
Number of neurological symptoms (range 0–5)	1.44 (1.40)	1.54 (1.49)	1.04 (1.36)	1.61 (1.43)	1.49 (1.48)	1.54 (1.41)	1.30 (1.39)	1.43 (1.45)	< 0.001
Number of digestive symptoms (0–3)	0.15 (0.48)	0.27 (0.60)	0.15 (0.46)	0.20 (0.52)	0.20 (0.53)	0.23 (0.56)	0.16 (0.48)	0.21 (0.54)	< 0.001
Number of respiratory and heart symptoms (range 0–4)	0.88 (0.98)	0.92 (1.10)	0.65 (0.92)	0.81 (1.05)	0.81 (1.05)	0.8 (0.97)	0.84 (1.02)	0.84 (1.04)	< 0.001
Number of other symptoms (range 0–3)	0.33 (0.58)	0.40 (0.66)	0.25 (0.52)	0.34 (0.62)	0.33 (0.60)	0.38 (0.64)	0.31 (0.58)	0.35 (0.62)	< 0.001
Number of post COVID-19 symptoms (range 0–15)	2.8 (2.58)	3.13 (3.01)	2.09 (2.52)	2.96 (2.65)	2.82 (2.80)	2.95 (2.74)	2.61 (2.60)	2.82 (2.80)	< 0.001

Table 4 revealed the brief model to identify factors associated with the number of four main types of post-COVID-19 symptoms (the full model was presented in Appendix 4). Compared to male participants, the female was likely to have a lower number of neurological symptoms, a lower number of other symptoms, but they had a higher number of digestive symptoms. Compared to people living in the Southeast region, people living in the Red River Delta were likely to have a lower number of neurological and respiratory, and heart symptoms, people living in North Central Region and South Central Coast also had a lower number of respiratory and heart symptoms, people who lived in South Central Coast and Mekong Delta Region was likely to have a higher number of digestive symptoms. Smoking and Drinking alcohol were the positive factors that increased the number of other symptoms and neurological symptoms, respectively. Overweight/obese people had a lower number of neurological symptoms but had a higher number of respiratory and heart symptoms and other symptoms.

**Table 4:** Brief Multilevel linear random-effects models to identify factors associated with neurological, digestive, respiratory, heart, and other symptoms.

Factors	Neurological symptoms		Digestive symptoms		Respiratory and heart symptoms		Other symptoms	
	Coef	*p*-value	Coef	*p*-value	Coef	*p*-value	Coef	*p*-value
Socio-economic								
Gender (Female vs Male -ref)	−0.35	< 0.001	0.06	< 0.001			−0.19	< 0.001
Age (unit: age)			−0.002	0.005				
Provinces (Southeast region -ref)								
Red River Delta	−0.28	0.023			−0.16	0.019		
North Central Region					−0.15	0.029		
South Central Coast			0.06	0.005	−0.11	0.023		
Mekong Delta Region			0.07	0.001				
Smoking (Yes vs No -ref)							0.06	0.002
Alcohol (Yes vs No -ref)	0.13	< 0.001						
BMI Index (vs Underweight -ref)								
Normal					0.06	0.007		
Overweight/Obese	−0.13	< 0.001			0.08	0.018	0.03	0.041
Comorbidities (Yes vs No -ref)					0.24	< 0.001		
COVID-19 infection characteristics								
COVID-19 infection period (vs Less than 7 days -ref)								
7–14 days	0.12	< 0.001	−0.01	0.048	0.06	0.002	0.03	0.011
More than 14 days	0.20	0.011	0.06	0.03	0.15	0.003	0.09	0.001
Time since COVID-19 onset (vs 1 month -ref)								
1–4 months	0.12	0.001	−0.04	0.001	−0.06	0.007		
4–6 months	0.13	0.023					−0.04	0.033
Severity of COVID-19 at the onset (vs asymptomatic -ref)								
Mild	0.26	< 0.001			0.17	< 0.001		
Moderate	0.56	< 0.001	0.07	0.004	0.40	< 0.001	0.10	< 0.001
Severe					0.39	0.001		
Prevalence of case in region (vs Low -ref)								
Medium			0.03	0.038				
Post-COVID-19 symptoms								
Neurological symptoms	–	–	0.07	< 0.001	0.25	< 0.001	0.12	< 0.001
Digestive symptoms	0.40	< 0.001	–	–	0.38	< 0.001	0.20	< 0.001
Respiratory and heart symptoms	0.43	< 0.001	0.12	< 0.001	–	–	0.07	< 0.001
Other symptoms	0.55	< 0.001	0.16	< 0.001	0.18	< 0.001	–	–

People who were infected with COVID-19 for 7–14 days or more than 14 days were likely to have a higher number of neurological symptoms, respiratory and heart symptoms, and other symptoms, but those who were infected with COVID-19 from 7 to 14 days had a lower number of digestive symptoms than those who infected with COVID-19 less than 7 days. People who recovered from COVID-19 for 1–4 months were likely to have a higher number of neurological symptoms, have a lower number of digestive and respiratory, and heart symptoms, but those who recovered from COVID-19 from 4 to 6 months had a higher number of neurological symptoms and a lower number of other symptoms. Compare to asymptomatic COVID-19 at the onset, people had mild severity had a higher number of neurological and respiratory, and heart symptoms. Moreover, people had moderate severity had a higher number of both of the four main symptoms. An increase in any one type of symptom was likely to increase the number of other symptoms.

Table 5 presented that female participants tended to experience lower negative impacts of COVID-19 such as lower severity of COVID-19 at the onset, lower level of post-COVID-19 symptoms, and less number of post-COVID-19. These characteristics also correlated with age, as younger people had a lower level of severity of COVID-19 at the onset, and fewer post-COVID-19 symptoms. Smoking, drinking alcohol, and having comorbidities were risk factors that were highly associated with the increase in the level of post-COVID-19 symptoms and the number of post-COVID-19 symptoms.

**Table 5:** Factors associated with severity of COVID-19 at the onset and post-COVID-19 symptoms.

Factors	Severity of COVID-19 at the onset (From 1″Asymptomatic” to 4″Severe”			Post COVID-19 symptom (From 0 “Asymptomatic” to 4 “4 or more than 4 symptoms”)			Number of Post COVID-19 symptoms		
	OR	95%CI	*p*-value	OR	95%CI	*p*-value	Coef	95%CI	*p*-value
Socio-economic									
Gender (Female vs Male -ref)	0.53	0.47; 0.61	< 0.001	0.41	0.39; 0.44	< 0.001	−1.14	−1.25; −1.03	< 0.001
Age (unit: age)	0.99	0.98; 1.00	0.025	0.99	0.98; 1.00	0.169	−0.03	−0.04; −0.01	< 0.001
Provinces (Southeast region -ref)									
Northern Midlands and Mountains	1.21	0.68; 2.16	0.519	0.73	0.51; 1.05	0.092	−0.20	−0.63; 0.23	0.370
Red River Delta	0.55	0.30; 0.98	0.043	0.34	0.16; 0.72	0.005	−1.15	−1.64; −0.65	< 0.001
North Central Region	1.46	0.89; 2.40	0.134	0.81	0.60; 1.08	0.155	−0.10	−0.46; 0.27	0.613
South Central Coast	1.08	0.70; 1.66	0.744	0.84	0.68; 1.04	0.103	0.04	−0.24; 0.33	0.763
Central Highlands	1.28	0.80; 2.05	0.308	0.78	0.47; 1.31	0.355	0.02	−0.35; 0.39	0.903
Mekong Delta Region	1.41	0.97; 2.04	0.069	0.84	0.70; 1.02	0.074	−0.03	−0.27; 0.21	0.814
Smoking (Yes vs No -ref)	0.73	0.57; 0.92	0.007	1.20	1.02; 1.42	0.029	0.23	0.00; 0.45	0.046
Alcohol (Yes vs No -ref)	1.21	1.05; 1.39	0.007	1.43	1.32; 1.55	< 0.001	0.44	0.34; 0.54	< 0.001
Exercising after recovering from COVID-19 (Yes vs No -ref)	0.91	0.79; 1.06	0.217	0.83	0.73; 0.95	0.008	−0.15	−0.34; 0.04	0.115
BMI Index (vs Underweight -ref)									
Normal	1.06	0.95; 1.18	0.320	1.01	0.93; 1.09	0.879	0.03	−0.09; 0.15	0.671
Overweight/Obese	0.82	0.66; 1.03	0.088	1.05	0.89; 1.24	0.572	0.03	−0.13; 0.18	0.716
Comorbidities (Yes vs No -ref)	1.95	1.32; 2.89	0.001	1.92	1.54; 2.40	< 0.001	1.04	0.68; 1.40	< 0.001
Infected with COVID-19 characteristic									
COVID-19 infection period (vs Less than 7 days -ref)									
7–14 days	3.15	2.87; 3.45	< 0.001	1.42	1.31; 1.53	< 0.001	0.50	0.40; 0.60	< 0.001
More than 14 days	14.04	10.27; 19.19	< 0.001	2.04	1.45 2.87	< 0.001	1.37	0.91; 1.83	< 0.001
Time since COVID-19 onset (vs 1 month -ref)									
1–4 months	0.90	0.82; 0.98	0.019	0.99	0.91; 1.08	0.837	−0.04	−0.2;1 0.12	0.619
4–6 months	0.58	0.50; 0.68	< 0.001	1.10	0.89; 1.37	0.359	0.15	−0.10; 0.39	0.240
Above 6 months	0.28	0.22; 0.38	< 0.001	0.84	0.73; 0.96	0.013	−0.19	−0.42; 0.04	0.105
Severity of COVID-19 at the onset (vs asymptomatic -ref)									
Mild	–	–	–	2.51	2.18; 2.89	< 0.001	1.01	0.76; 1.26	< 0.001
Moderate	–	–	–	7.30	5.91; 9.02	< 0.001	2.91	2.62; 3.19	< 0.001
Severe	–	–	–	3.89	2.36; 6.43	< 0.001	2.39	1.54; 3.24	< 0.001
Prevalence of case in region (vs Low -ref)									
Medium	1.23	0.95; 1.60	0.113	0.94	0.77; 1.15	0.544	0.10	−0.05; 0.25	0.187
High	2.29	1.43; 3.66	0.001	1.48	0.87; 2.51	0.144	0.60	0.19; 1.02	0.004
COVID-19 case fatality rate in region (vs Low -ref)									
Medium	0.89	0.58; 1.37	0.596	0.79	0.49; 1.25	0.311	−0.28	−0.64 0.09	0.135
High	0.81	0.48; 1.36	0.420	0.68	0.41; 1.13	0.137	−0.48	−0.91; −0.05	0.028

In this study, the Southeast region was chosen as the reference factor. Southeast Region included Ho Chi Minh, Ba Ria—Vung Tau, Binh Duong, Binh Phuoc, Dong Nai, and Tay Ninh, which were heavily affected by COVID-19. Especially, Ho Chi Minh City and Binh Duong are the two epicenters of the country's outbreak of the COVID-19 pandemic with the highest number of cases and deaths^21^. The Vietnamese government has many policies to help reduce the damage of COVID-19 such as establishing a mobile medical station, and mobilizing a large force of health workers, the army, and the police to participate in epidemic prevention and control^22^. Therefore, choosing the Southeast region as a reference helps to objectively compare the severity of COVID-19 at the onset and post-COVID-19 symptoms and provides more information for readers and policymakers. Compared to participants living in the Southeast Region, people who resided in the Red River Delta had a lower level of severity of COVID-19 at the onset, lower level of post-COVID-19 symptoms, and less number of post-COVID-19 symptoms.

Longer infection time of COVID-19 and higher severity of COVID-19 at the onset were associated with an increase in both levels of post-COVID-19 symptoms and the number of post-COVID-19 symptoms. People who lived in the region with a high prevalence of case was likely to have a higher severity of COVID-19 at the onset and more number post-COVID-19 symptoms.

Appendix 1, Appendix 2, and Appendix 3 revealed post-COVID-19 symptoms and the number of post-COVID-19 symptoms by COVID-19 characteristics.

**Figure 2:**
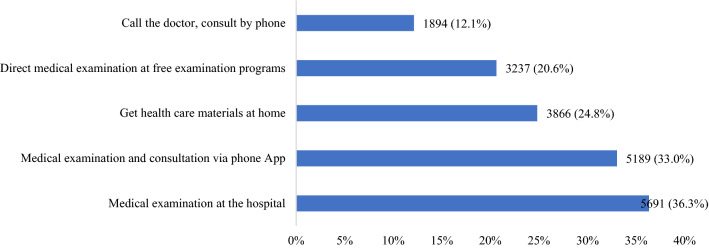
Preference of post-COVID-19 care among respondents.

Figure 2 presents the preference of post-COVID-19 care among respondents. The most favorable means of care delivery among respondents was directly at hospitals (36.3%) or through mobile apps (33%). Medical consultation through telephone received the lowest preference level 12.1%).

## Discussion

Our results suggested a high frequency and severity level of post-COVID-19 symptoms among Vietnamese and identified age, infection duration, daily health behavior, and comorbidities as factors associated with the prevalence and severity of post-COVID-19 symptoms. Multilevel implications were proposed, including clinical development as well as adjustments to health policies and government management strategy.

Correlations were found between demographic, behavioral factors, and post-infection symptoms. Our data indicated a strong association between gender and post-COVID-19 symptoms, in which females tended to experience less severe chronic COVID-19 syndromes than males. This may result from sex differences in immune response^23^. While some existing studies recorded similar results, others found an opposite pattern of gender severity, such as the longitudinal cohort study in Germany or a cross-sectional study of healthcare workers in Northwest England, where females experienced more severe post-COVID-19 manifestations^24–26^. Therefore, although findings suggest that gender may not be a significant determinant of post-COVID-19 conditions, more empirical evidence should be collected to determine this correlation. Interestingly, the number of post-COVID-19 conditions decreased with age, meaning older participants recorded fewer symptoms than young participants. Although no previous study has focused on age as a determinant, this finding may underline the need to encourage post-COVID-19 care among the younger population. Overweight and comorbidities were found to correlate with a heightened likelihood of developing post-COVID-19 symptoms in neurological and respiratory systems. Indeed, problems with nutrition levels are determinants of cardiovascular disease and cancer, the two most common comorbidities among patients with severe infection and longer post-infection syndromes in our results^27–29^. As these dynamics have been observed in the majority of existing literature, this study reaffirms the importance of practicing regular healthy activities as well as highlights a novel risk of alcohol and smoking^30–32^.

Longer infection duration and severity were correlated with substantially higher severity and several post-COVID-19 symptoms. Our findings demonstrated that infection duration of more than 14 days and moderate severity were most correlated with a higher number of symptoms as well as higher severity. While this trend is neither refuted nor contrasted in any existing studies, the strength of such association varies: from substantial in our findings, briefly discussed in Hall’s et al. to insignificant or not mentioned at all in many^31,33,34^. The gap in data may be attributed to disparities between the healthcare system and the socioeconomic background of each sample. A more reasonable explanation lies in the difference between assessment scales and definitions of post-infection symptoms between studies. Baig et al. proposed that symptoms prolonging beyond 3 weeks be classified as chronic COVID-19 symptoms, while another study proposed the categorization for different post-infection durations: post-acute COVID-19 for symptoms beyond 3 weeks and long post-COVID-19 for symptoms beyond 12 weeks^35,36^.

Our findings and previous meta-analysis worldwide pointed out that the most frequent post-infection symptoms were related to the neurological and respiratory systems, such as dizziness, headache, cough, or shortness of breath. The multivariate random effect models demonstrated an important pattern that post-COVID-19 symptoms tended to co-exist across systems. The presence of either one of three symptom groups: neurological, digestive, and respiratory, was significantly associated with the presence of the other two. To date, the approach of pharmaceutical treatments for post-COVID-19 symptoms tends to focus on each symptom at a time instead of finding a solution for the multisystem manifestation of post-COVID-19. If clinically possible, interventions for post-COVID-19 symptoms should be developed to resolve multisystem problems instead of focusing on one single symptom. Furthermore, although these manifestations can cause great inconveniences to daily functioning and result in lower quality of life for recovered patients, they are generally mild and rarely develop into severe complications^37–39^. However, such symptoms are also indicators of many serious, even fatal, health conditions. For example, chronic migraines suggest the possible onset of a cerebrovascular accident or a brain tumor, while constant coughs can indicate early stages of gastroesophageal reflux disease or pulmonary tuberculosis^40–43^. The misperception of these mild conditions as post-COVID-19 symptoms prevents individuals from having an early diagnosis and ultimately leads to poor treatment outcomes. Therefore, besides developing pharmaceutical treatments for chronic COVID-19, recovered patients should be reminded of potential misunderstandings and be informed about how to distinguish post-COVID-19 symptoms from early manifestations of other serious health conditions.

Most notably, the proportion of post-COVID-19 symptoms varies across regions and is attributable to differences in socioeconomic characteristics, health delivery, and level of adaptation to response strategy. The severity of post-COVID-19 symptoms nearly doubled in regions with a high prevalence of cases compared to those with low prevalence. In Vietnam, the most notable differences can be observed in the Red River Delta (RRD) and the Northern Midlands and Mountains (NMM). Residents in RRD recorded lower numbers and severity of post-COVID-19 conditions than all other regions. Moreover, while the largest proportion of participants in other regions had 4 or more post-COVID-19 symptoms, most participants from RRD were asymptomatic (33.4%) or had one symptom (20.3%). On the other hand, residents from NMM had both the highest number and highest severity of post-COVID-19 symptoms than the remaining provinces. The pattern observed in RRD and NMM can be attributed to gaps in healthcare quality and the health literacy of residents. The RRD is the region with the highest population density in Vietnam (1,450 people/km^2^, the population is 21,848,913 people), is the driver of Vietnam’s industrial growth as one of the most developed, populated regions of Vietnam and consists of the capital Hanoi^44,45^. RRD accounts for 29.4% of Vietnam's GDP and the monthly average income per capita in 2020 in RRD was 5.005 million VND (~ $210)^46,47^. Understandably, most resources are directed toward this region, resulting in a highly trained healthcare workforce, better health access, and consequently better awareness of post-COVID-19 conditions among residents^48^. By contrast, NMM is among the most sparsely populated and underinvested regions of Vietnam. NNM is a region with a GDP equal to 1/3 of RRD (8.54% of Vienam's GDP)^49^, monthly average income per capita in 2020 was 2745 million VND (~ $115)^46^, and received the least healthcare attention during COVID-19^50,51^. Vietnam has a serious shortage of healthcare workers, which becomes more serious when the COVID-19 pandemic occurs. Specifically, to influence the impact of the COVID-19 epidemic, there were 9,680 health workers who resigned or quit^52^, leading to NNM being heavily affected. To now, only 19.9% of residents in Son La received the 3^rd^ dose of the COVID-19 vaccine^53^.

The case of RRD and NMM suggested that COVID-19 awareness and care-seeking intention play a crucial role in reducing post-COVID-19 conditions. The adaptability to national response also contributes to the inconsistency of COVID-19 outcomes across regions. During the peak of the pandemic, NMM recorded a relatively lower prevalence of cases compared to RDD and other metropolitan areas^51^. However, low infection rates in NMM were primarily due to secluded locations, rather than successful pandemic control and compliance of residents. On the other hand, while being the center of coronavirus spread and suffering the most devastating damages in terms of human, financial capacity and health workforce, RDD is among regions with the highest levels of adaptability to national guidelines such as social distancing, masking, and m-health utilization. As post-COVID-19 symptoms are long-term and rarely fatal, the distinguishing factor is adaptability to the national response. Differences in levels of awareness and compliance to guidelines between RDD and NMM demonstrate a clear correlation to post-COVID-19 outcomes. In this post-COVID-19 era, it is important that we consider levels of adaptation to previous strategies as the key indicator instead of the number of cases or deaths in a population.

Regarding post-COVID-19 care, 33% of patients chose mobile applications as their preferred healthcare platform compared to 36.3% who preferred traditional hospital appointments. This number showed substantial progress in the transformation of healthcare delivery in Vietnam from traditional to online platforms. Before COVID-19, online healthcare models were not popular nor encouraged in Vietnam due to inconsistencies in care quality between national, public facilities, and online services at the current time. In 2020, social restrictions due to COVID-19 have not only served as an incentive for citizens to utilize telehealth services, but also promoted the improvement of care among providers to cater to the increasing needs of the population, such as the national hotline to resolve inquiries about COVID-19 at home, Zalo COVID-19 consultation chatbot or televised and online art therapy sessions by non-profit organizations to name a few^54–57^. After 2 years of COVID-19, it is clear that Vietnamese are adapting to the telehealth model, and telehealth promotion has also identified as one of Vietnam’s digital transformation goals from 2020 to 2025 under project 2628/QDBYT. Therefore, it is important that this favorable trend continues even after COVID-19 restrictions are lifted.

Several implications can be drawn from the above findings. First, a global framework should be provided, including non-COVID variables such as age, daily health behavior, and comorbidities. In this study, these terms were used as one under the term “post-COVID-19 symptoms” and defined as symptoms presenting for more than 4 weeks. Various terms for post-COVID-19 symptoms such as “long-COVID”, “chronic COVID” or “post-acute COVID-19” should be distinguished with specific time periods. More data on post-COVID-19 conditions should be collected from resource-scarce settings and in LMICs to inform evidence-based interventions and identify multidimensional determinants such as social, financial, and cultural characteristics of each population. Secondly, validated information should be provided more extensively to raise awareness of post-COVID-19 symptoms and avoid potential confusion between post-COVID-19 conditions and symptoms of other diseases. When developing and applying a national strategy for post-COVID-19 conditions, contextual and behavioral characteristics of different settings such as health system capacity, resident behavior, and level of adaptation to guidelines must be considered. At the managerial level, healthcare resources should be distributed more evenly to ensure health access and adequate information in underserved regions, such as the Northern Midlands and Mountains. As these regions are scarce in resources, instead of large-scale events, province leaders can utilize television and social media as effective means of information delivery to improve health literacy, and address hesitancy toward care-seeking practices among residents. In terms of rehabilitation, it is important that post-COVID-19 care be provided regularly for a length of time due to the long-term nature of this condition. We suggest that existing resources be mobilized and managed by regions or communities instead of the government to ensure a rapid response. As discussed, adaptability to national strategy varies across regions, and thus it may be more effective for authorities to decide on best practices for their populations. Finally, policymakers should strengthen digital safety and privacy of healthcare consulting through technology platforms as well as empower youth-led digital healthcare startups in delivering health services to relieve the burden of the national frontline.

Our study's sample size is a strength, as it was drawn from one of the largest post-COVID-19 care initiatives in Vietnam, providing a national reference dataset. However, there are several limitations to our findings. Firstly, because this was a cross-sectional study, we cannot infer causation. Secondly, our data may be under-reported due to the recruitment of participants only from health facilities, which excludes those who did not seek medical attention despite experiencing post-COVID-19 symptoms. This exclusion disproportionately affects older adults. Additionally, our study focused on the young workforce, recognizing their importance in national development, and as a result, the majority of responses were obtained from this age group. Thirdly, it is essential to note that COVID-19 grading criteria were self-reported, which could be subject to recall bias. Therefore, we combined medical records with participants' responses to minimize this potential bias. Fourthly, there may be selection biases resulting from the online survey platform, which relied on volunteers to guide eligible participants through the questionnaire. Lastly, our study only examined the 15 most common post-COVID-19 symptoms, and other less common symptoms were not included. Nonetheless, our findings identified concerning trends in post-COVID-19 symptoms and have significant implications for policymakers, clinicians, and the general public.

Besides, it is important to acknowledge that our sample might be affected by a potential selection bias and decreased representativeness given that two-thirds of respondents were male and the median age was 18. Previous studies have shown sex differences in sex outcomes, thus, our findings might not fully representative of the whole Vietnamese population from an epidemiological view. However, from an evidence-informed policy making perspective, we would emphasize that this is the largest and most optimal sample we could recruit in the Vietnamese population, in a timely manner. First, because of the confidentiality of the information regarding COVID-19 patients, it is impossible to construct a nationally representative sample frame and sample. Secondly, for resource-scare settings, the implementation of free-of-charge health check-ups on this large scale is unique, especially during the COVID-19 pandemic. Last but not least, the timing of evidence ready to inform policy development in Vietnam that this study has provided is a good example of knowledge translation, and could serve as a reference for global health policy practice.

## Conclusion

Our study investigates the prevalence of post-COVID-19 symptoms among recovered patients in Vietnam. A gap in definitions should be addressed before developing a timeframe and response strategy for chronic COVID-19. We were able to identify several determinants, including age, infection period, and underlying conditions as well as an important trend in telehealth utilization. Disproportionate distribution of resources and inconsistent post-COVID-19 patterns were found across regions, which suggested a shift in strategy implementation from national to province-based. In the upcoming phase in the fight against COVID-19, it is important that post-COVID-19 symptoms are monitored closely and tackled by a combination of pharmaceutical, psychological, and public health interventions.

## Methods

### Study design and sampling methods

On May 2022, a cross-sectional study was conducted on participants who registered for post-COVID-19 medical examination during the Post-COVID-19 Care Program in accordance with Plan No. 52-KH/TWH of the Central Committee of the Vietnam Youth Union. This government program focused on post-COVID-19 health care for the Vietnamese population. Those who come to examination include people who had COVID-19 and were experiencing post-COVID-19 symptoms as listed by the Ministry of Health.

The research team developed a survey questionnaire consisting of 4 parts and 20 questions online. When people participate in Post-COVID-19 Care Program, volunteers have guided participants by scanning the QR code which allowed people access directly to the questionnaire. Volunteers were issued around hospitals and health examination points to guide participants in completing the questionnaire. The severity of COVID-19 at the onset of participants was collected by volunteers from medical records for patients with COVID-19.

### Participants

All people infected with COVID-19 and who had medical records for patients with COVID-19 were invited to participate in the study. There were 49,496 patients are examined and consulted in the Post-COVID-19 Care Program, 17,093 individuals returned to this survey, and 12,361 completed the survey. The completion rate was 72.3%.

The criteria for selecting the subjects were as follows:

1. Aged from 16 to 35 years old
2. Agree to participate in the study
3. Took part in medical examination during the Post COVID-19 Care Program
4. Currently living in Vietnam

Exclusion criteria:

1. Suffered from serious cognitive impairment or were unable to answer questions
2. Were unable internet access, survey link, and reach out and complete surveys

### Measurements

A structured questionnaire was developed consisting of three main components: (1) Individual characteristics; (2) Characteristics of participants when infected with COVID-19, and (3) Post-COVID-19 symptoms characteristics. The questionnaire was first uploaded to the online survey platform SurveyMonkey (surveymonkey.com), a convenient and cost-effective survey design platform that enables quick, efficient data collection, especially in times of social distancing. This approach may exclude certain population groups in Vietnam who do not have access to smart devices, but smartphone usage in Vietnam ranked top 10 globally^58^. By March 2022, the total number of smartphone subscribers was 93.5 million, reaching 73.5% of the country’s adults^59^. Hence, it is reasonable to use this approach in the COVID-19 context in Vietnam.

The structured questionnaire was then finalized with the following main sections:

## Variables

### Outcome variables

#### Post-COVID-19 symptoms and the number of post-COVID-19 symptoms

We designed 15 items corresponding to 15 common symptoms during COVID-19 recovery, including rash, diarrhea, change in smell or taste, abdominal pain, chest pain, fast beating, anxiety, changes in menstrual cycles, myalgia, somnipathy, dyspnea, cough, difficulty thinking or concentrating, headache, and fatigue. Participants were then divided into 5 groups, including 0 “Asymptomatic”; 1 “1 symptom”; 2 “2 symptoms”; 3 “3 symptoms”; and 4 “4 or more than 4 symptoms”. We also calculated the number of post-COVID-19 symptoms of participants and categorized them as neurological symptoms, digestive symptoms, respiratory and heart symptoms, or other symptoms through 15 common symptoms of patients. It is vital to note that not only is symptom presence required but so are symptom duration and intensity^60^. Hence, we used the number of post-COVID-19 symptoms as a variable to measure the severity of post-COVID-19 symptoms.

*Neurological symptoms*: fatigue, difficulty thinking or concentrating, headache, somnipathy, and anxiety. The number of neurological symptoms ranged from 0 to 5.

*Digestive symptoms*: change in smell or taste, diarrhea, and abdominal pain. The number of digestive symptoms ranged from 0 to 3.

*Respiratory and heart symptoms*: dyspnea, cough, chest pain, fast beating. The number of respiratory and heart symptoms ranged from 0 to 4.

*Other symptoms:* rash, myalgia, and changes in menstrual cycles. The number of other symptoms ranged from 0 to 3.

### Covariate

#### Individual characteristics

Respondents reported their socio-demographic information, including age, gender (male/female), BMI index, and the city they lived in, which were divided into seven economic zones of Vietnam: Southeast Region, Northern Midlands and Mountains, Red River Delta, North Central Region, South Central Coast, Central Highlands, and Mekong Delta Region. Map of 63 provinces in Vietnam was presented in Appendix 6.

#### Behaviors

Participants self-reported their current status of smoking, alcohol usage, and exercise after recovering from COVID-19.

#### Co-morbidities

Participants reported their comorbidities which were diagnosed by doctors. There were some common comorbidities such as diabetes, cardiovascular disease, chronic lung disease, neurological disease, and cancer.

### Characteristics of COVID-19 infection

We used three questions to measure the characteristics of COVID-19 infection among participants, including:

#### Time since COVID-19 onset

1 month, 1–4 months, 4–6 months, and above 6 months. Time since COVID-19 onset as a period between the date when COVID-19 patients were confirmed negative for COVID-19 using PCR tests to the time they entered this study.

#### COVID-19 infection period

Less than 7 days, 7–14 days, and more than 14 days. COVID-19 infection period was defined as a period between when people were confirmed positive for COVID-19 and when they were confirmed negative for COVID-19 using the PCR test.

#### The Severity of COVID-19 at the onset

Asymptomatic, mild, moderate, and severe^61^:

- *Asymptomatic* testing positive for COVID-19 but having no symptoms that are consistent with COVID-19 infection.
- *Mild* having any of the following signs and symptoms of COVID-19. fever, cough, sore throat, malaise, headache, muscle pain, nausea, vomiting, diarrhea, loss of taste and smell; but not having shortness of breath, dyspnea, or abnormal chest imaging.
- *Moderate* experiencing lower respiratory disease during clinical assessment or imaging and having SpO2 above 94% on room air at sea level.
- *Severe* having SpO2 below 94% on room air at sea level, PaO2/FiO2 < 300 mm Hg, respiratory rate above 30 breaths/min, or lung infiltrates above 50%.

The severity of COVID-19 at the onset of the participant was diagnosed by the doctor and divided into four levels: asymptomatic, mild, moderate, and severe.

### Prevalence of COVID-19 cases by region

The total number of COVID-19 was taken from official data of the Ministry of Health as of June 14, 2022^62^. We measured the prevalence of COVID-19 cases per 100,000 population and the case fatality rate due to COVID-19 using the following formula:

$$\begin{gathered} Prevalence \, of \, COVID - 19 \, cases \, per \, 100,000 \, population\;in\;a\;region\, \hfill \\ = \,(Number \, of \, COVID - 19 \, cases\;in\;a\;region)/(Number \, of \, population\;in\;a\;region)\, \times \,100,000. \hfill \\ \end{gathered}$$

$$\begin{gathered} {\text{Case fatality rate}}\, = \,(Number \, of \, deaths \, due \, to \, COVID - 19\;in\;a\; \hfill \\ region)/(Number \, of \, COVID - 19 \, cases\;in\;a\;region)\, \times \,100. \hfill \\ \end{gathered}$$

After that, we divided the prevalence of COVID-19 cases per 100,000 population and the case fatality rate due to COVID-19 into three groups low, medium, and high prevalence.

Data on the number of COVID-19 cases, the number of deaths due to COVID-19, the prevalence of COVID-19 cases, and the case fatality rate due to COVID-19 was presented in Appendix 1.

### Preference of post-COVID-19 care

Participants self-reported their preference for health care and consult after recovering from COVID-19, including medical examination at the hospital, medical examination, and consultation via phone App, getting health care materials at home, direct medical examination at free examination programs, calling the doctor, and consulting by phone.

### Data analysis

We analyzed data using STATA version 16 (Stata Corp. LP, College Station, United States of America). With missing data, we used the Listwise Deletion method to clean data before analyzing. Continuous variables were presented as mean and standard deviation (SD), while categorical variables were presented as frequencies with percentages. We used the Chi-squared and Kruskal–Wallis tests to test the difference between each group. We used the “xtile” function to separate the prevalence of COVID-19 cases and the case fatality rate due to COVID-19 into 3 groups: low, medium, and high prevalence.

A multi-level modeling approach with mixed effect model was used for determining associated factors of post-COVID-19 severity at the regional level while adjusting for differences in provincial policies and regulations on COVID-19 control and preparedness which were treated as random effects.

Potential covariates for full models of four main types of post-COVID-19 symptoms, COVID-19 at the onset, level of post-COVID-19 symptoms, and the number of post-COVID-19 symptoms included individual characteristics, clinical manifestations when the got infected with COVID-19, the prevalence of COVID-19 cases, and case-fatality rate due to COVID-19. We used multilevel linear random-effects models to identify the factor associated with the number of post-COVID-19 symptoms. Multi-level random effects ordered logistic model was used to identify the factors related to the severity of COVID-19 at the onset and the level of post-COVID-19 symptoms. In this study, we used location which includes 63 provinces in Vietnam as a cluster variable. A *p*-value (P) of < 0.05 was considered statistically significant.

### Ethical approval

Ethical approval is granted by Hanoi Medical University and Youth Research Institute; the research protocol was reviewed by the Vietnam Young Physician Association and the Institute of Health Economics and Technology’s scientific committee. All participants had provided informed consent, and the survey did not have any impact on their medical examination and consultation process. Study data were de-identified for analysis. All procedures performed in studies involving human participants were in accordance with the ethical standards of the 1964 Helsinki Declaration and its later amendments or comparable ethical standards. Patients invited to participate in the study have fully explained the content, purpose, and benefits when participating in the study. The collected information is kept confidential and the information is only for research purposes, not for other purposes, the data is encrypted to ensure confidentiality of the information. Participants can refuse to participate in the study at any time. During the participation process, they can still withdraw from the study if the research they feel uncomfortable.

## Supplementary Information

Supplementary Information 1.

Supplementary Information 2.

Supplementary Information 3.

Supplementary Information 4.

Supplementary Information 5.

Supplementary Information 6.

## Data Availability

The datasets used and/or analyzed during the current study are available from the corresponding author on reasonable request.
