# Supplementary material for: Severity and geographical disparities of post-COVID-19 symptoms among the Vietnamese general population: a national evaluation
Source: Sci Rep. 2023 Mar 17;13:4460. doi: 10.1038/s41598-023-30790-x (PMC10022561; doi:10.1038/s41598-023-30790-x)
Supplement: Supplementary file 1 — Supplementary Information 1. [file 41598_2023_30790_MOESM1_ESM.pdf]

Appendix 1. Characteristics of 4 symptoms of post-COVID-19 symptoms by COVID-19 characteristics

Characteristics	Neurological symptoms			Digestive symptoms			Respiratory and heart symptoms			Other symptoms		
	n	%	p-value	n	%	p-value	n	%	p-value	n	%	p-value
**Time since COVID-19 onset**												
1 month	1098	15.3	<0.001	268	18.8	<0.001	1009	16.1	< 0.001	540	15.9	< 0.001
1-4 months	5168	71.8		969	67.9		4396	70.2		2470	72.6	
4-6 months	525	7.3		92	6.4		482	7.7		218	6.4	
Above 6 months	402	5.6		98	6.9		372	5.9		174	5.1	
**COVID-19 infection period**												
Less than 7 days	3229	44.8	<0.001	619	43.2	<0.001	2811	44.8	< 0.001	1419	41.6	< 0.001
7-14 days	3795	52.7		754	52.7		3294	52.5		1891	55.5	
More than 14 days	180	2.5		59	4.1		167	2.7		99	2.9	
**Severity of COVID-19 at the onset**												
Asymptomatic	437	6.1	<0.001	74	5.2	<0.001	386	6.1	< 0.001	168	4.9	< 0.001
Mild	5864	81.2		1086	75.7		5056	80.4		2690	78.8	
Moderate	861	11.9		254	17.7		790	12.6		515	15.1	
Severe	60	0.8		21	1.5		53	0.8		40	1.2	
	**Mean**	**SD**	**p-value**	**Mean**	**SD**	**p-value**	**Mean**	**SD**	**p-value**	**Mean**	**SD**	**p-value**
**Time since COVID-19 onset**												
1 month	1.09	1.24	<0.001	0.18	0.48	0.001	0.88	1.06	0.001	0.36	0.63	< 0.001
1-4 months	1.14	1.24		0.14	0.42		0.84	1.03		0.36	0.63	
4-6 months	1.08	1.26		0.13	0.43		0.86	1.06		0.28	0.56	
Above 6 months	0.84	1.17		0.15	0.44		0.74	1.06		0.25	0.54	
**COVID-19 infection period**												
Less than 7 days	0.94	1.15	<0.001	0.13	0.41	<0.001	0.71	0.97	< 0.001	0.28	0.56	< 0.001
7-14 days	1.27	1.28		0.16	0.46		0.95	1.09		0.41	0.66	
More than 14 days	1.67	1.51		0.31	0.61		1.33	1.25		0.58	0.81	
**Severity of COVID-19 at the onset**												
Asymptomatic	0.50	0.89	<0.001	0.07	0.32	<0.001	0.38	0.71	< 0.001	0.15	0.44	< 0.001
Mild	1.10	1.20		0.14	0.42		0.82	1.01		0.33	0.59	
Moderate	1.90	1.44		0.32	0.62		1.50	1.27		0.67	0.82	
Severe	1.88	1.63		0.33	0.61		1.49	1.49		0.67	0.80	
