# Supplementary material for: Severity and geographical disparities of post-COVID-19 symptoms among the Vietnamese general population: a national evaluation
Source: Sci Rep. 2023 Mar 17;13:4460. doi: 10.1038/s41598-023-30790-x (PMC10022561; doi:10.1038/s41598-023-30790-x)
Supplement: Supplementary file 2 — Supplementary Information 2. [file 41598_2023_30790_MOESM2_ESM.pdf]

Appendix 2. Characteristics of post-COVID-19 symptoms by COVID-19 characteristics

Characteristics	Post COVID-19 symptom										p-value
	Asymptomatic		One symptom		Two symptoms		Three symptoms		Four or more than four symptoms		
	n	%	n	%	n	%	n	%	n	%	
**Time since COVID-19 onset**											
1 month	377	15.1	389	15.2	287	15.1	215	14.5	621	16.2	< 0.001
1-4 months	1695	67.7	1722	67.4	1349	71.0	1082	73.0	2729	71.4	
4-6 months	197	7.9	215	8.4	144	7.6	102	6.9	279	7.3	
Above 6 months	233	9.3	229	9.0	120	6.3	83	5.6	193	5.0	
**COVID-19 infection period**											
Less than 7 days	1523	60.7	1429	55.8	978	51.3	682	45.9	1530	40.0	< 0.001
7-14 days	961	38.3	1091	42.6	897	47.0	778	52.4	2171	56.7	
More than 14 days	25	1.0	41	1.6	32	1.7	25	1.7	125	3.3	
**Severity of COVID-19 at the onset**											
Asymptomatic	563	22.0	420	16.3	157	8.2	84	5.6	122	3.2	< 0.001
Mild	1919	75.0	2021	78.7	1624	85.0	1278	85.9	3016	78.6	
Moderate	71	2.8	110	4.3	120	6.3	121	8.1	652	17.0	
Severe	6	0.2	18	0.7	10	0.5	4	0.3	45	1.2	
