# Supplementary material for: Severity and geographical disparities of post-COVID-19 symptoms among the Vietnamese general population: a national evaluation
Source: Sci Rep. 2023 Mar 17;13:4460. doi: 10.1038/s41598-023-30790-x (PMC10022561; doi:10.1038/s41598-023-30790-x)
Supplement: Supplementary file 3 — Supplementary Information 3. [file 41598_2023_30790_MOESM3_ESM.pdf]

Appendix 3. Characteristics of number of post-COVID-19 symptoms by COVID-19 characteristics

Characteristics	Number of post-COVID-19 symptoms		
	Mean	SD	p-value
**Time since COVID-19 onset**			
1 month	2.90	2.87	< 0.001
1-4 months	2.87	2.78	
4-6 months	2.73	2.78	
Above 6 months	2.34	2.83	
**COVID-19 infection period**			
Less than 7 days	2.39	2.58	< 0.001
7-14 days	3.21	2.90	
More than 14 days	4.41	3.65	
**Severity of COVID-19 at the onset**			
Asymptomatic	1.30	1.99	< 0.001
Mild	2.77	2.65	
Moderate	4.99	3.41	
Severe	4.93	4.01	
