# Supplementary material for: Severity and geographical disparities of post-COVID-19 symptoms among the Vietnamese general population: a national evaluation
Source: Sci Rep. 2023 Mar 17;13:4460. doi: 10.1038/s41598-023-30790-x (PMC10022561; doi:10.1038/s41598-023-30790-x)
Supplement: Supplementary file 4 — Supplementary Information 4. [file 41598_2023_30790_MOESM4_ESM.pdf]

Appendix 4: Multilevel linear random-effects models to identify factors associated with neurological, digestive, respiratory, heart, and other symptoms

Factors	Neurological symptoms			Digestive symptoms			Respiratory and heart symptoms			Other symptoms		
	Coef.	95%CI	p-value	Coef.	95%CI	p-value	Coef.	95%CI	p-value	Coef.	95%CI	p-value
**Socio-economic**												
**Gender (Female vs Male -ref)**	-0.35	-0.39; -0.30	<0.001	0.06	0.04; 0.08	<0.001	0.03	-0.01; 0.07	0.16	-0.19	-0.21; -0.18	< 0.001
**Age (unit: age)**	-0.01	-0.01; 0.00	0.134	-0.002	-0.004; -0.01	0.005	-0.003	-0.008; 0.001	0.145	0.00	-0.002; 0.002	0.933
**Provinces (Southeast region -ref)**												
Northern Midlands and Mountains	-0.06	-0.26; 0.13	0.513	0.06	-0.00; 0.12	0.064	-0.12	-0.26; 0.03	0.108	0.02	-0.05; 0.08	0.64
Red River Delta	-0.28	-0.52; -0.04	0.023	0.03	-0.04; 0.10	0.344	-0.16	-0.28; -0.03	0.019	-0.06	-0.13; 0.01	0.082
North Central Region	0.15	-0.00; 0.31	0.054	0.03	-0.01; 0.07	0.15	-0.15	-0.29; -0.02	0.029	-0.05	-0.10; 0.01	0.093
South Central Coast	0.08	-0.07; 0.24	0.30	0.06	0.02; 0.10	0.005	-0.11	-0.20; -.0.01	0.023	-0.02	-0.07; 0.02	0.305
Central Highlands	0.11	-0.05; 0.26	0.18	0.05	-0.01; 0.12	0.107	-0.15	-0.32; 0.02	0.076	0.00	-0.06; 0.05	0.848
Mekong Delta Region	-0.07	-0.20; 0.05	0.245	0.07	0.03; 0.11	0.001	-0.05	-0.14; 0.04	0.272	0.01	-0.04; 0.06	0.67
**Smoking (Yes vs No -ref)**	-0.01	-0.13; 0.11	0.872	0.02	-0.02; 0.07	0.35	-0.01	-0.11; 0.08	0.812	0.06	0.02; 0.10	0.002
**Alcohol (Yes vs No -ref)**	0.13	0.07; 0.19	<0.001	0.01	-0.02; 0.04	0.38	0.02	-0.03; 0.07	0.351	0.01	-0.02; 0.04	0.656
**Exercising after recovering from COVID-19 (Yes vs No -ref)**	-0.02	-0.06; 0.01	0.229	0.00	-0.01; 0.02	0.603	-0.02	-0.06; 0.03	0.442	-0.02	-0.06; 0.02	0.26
**BMI Index (vs Underweight -ref)**												
Normal	-0.06	-0.13; 0.00	0.069	0.00	-0.02; 0.02	0.989	0.06	0.02; 0.10	0.007	0.00	-0.02; 0.03	0.769
Overweight/Obese	-0.13	-0.21; -0.06	<0.001	0.01	-0.01; 0.03	0.456	0.08	0.01; 0.15	0.018	0.03	0.00; 0.06	0.041
**Comorbidities (Yes vs No -ref)**	0.13	0.03; 0.23	0.014	0.04	-0.05; 0.12	0.385	0.24	0.16; 0.32	<0.001	-0.01	-0.07; 0.05	0.777
**COVID-19 infection characteristics**												
**COVID-19 infection period (vs Less than 7 days -ref)**												

7-14 days	0.12	0.08; 0.16	<0.001	-0.01	-0.02; -0.00	0.048	0.06	0.02; 0.10	0.002	0.03	0.01; 0.05	0.011
More than 14 days	0.20	0.05; 0.35	0.011	0.06	0.01; 0.12	0.03	0.15	0.05; 0.25	0.003	0.09	0.03; 0.14	0.001
**Time since COVID-19 onset (vs 1 month -ref)**												
1-4 months	0.12	0.05; 0.19	0.001	-0.04	-0.06; -0.02	0.001	-0.06	-0.11; -0.02	0.007	0.00	-0.03; 0.04	0.976
4-6 months	0.13	0.02; 0.24	0.023	0.03	-0.02; 0.07	0.215	-0.04	-0.10; 0.01	0.135	-0.04	-0.07; -0.00	0.033
Above 6 months	-0.04	-0.12; 0.04	0.329	0.02	-0.00; 0.05	0.064	-0.03	-0.09; 0.03	0.326	-0.03	-0.08; 0.02	0.204
**Severity of COVID-19 at the onset (vs asymptomatic -ref)**												
Mild	0.26	0.19; 0.32	<0.001	0.00	-0.02; 0.02	0.848	0.17	0.14; 0.20	<0.001	-0.01	-0.04; 0.01	0.263
Moderate	0.56	0.44; 0.68	<0.001	0.07	0.02; 0.11	0.004	0.4	0.29; 0.52	<0.001	0.10	0.06; 0.14	< 0.001
Severe	0.28	-0.01; 0.56	0.056	0.13	-0.03; 0.29	0.116	0.39	0.17; 0.61	0.001	0.08	-0.08; 0.23	0.315
**Prevalence of case in region (vs Low -ref)**												
Medium	0.04	-0.04; 0.12	0.373	0.03	0.00; 0.07	0.038	-0.06	-0.14; 0.03	0.21	0.01	-0.01; 0.03	0.207
High	0.12	-0.08; 0.33	0.238	0.02	-0.05; 0.09	0.512	0.02	-0.12; 0.16	0.794	0.06	-0.01; 0.12	0.078
**COVID-19 case fatality rate (vs Low -ref)**												
Medium	-0.03	-0.18; 0.11	0.654	0.01	-0.04; 0.06	0.764	-0.11	-0.24; 0.03	0.116	0.02	-0.04; 0.07	0.578
High	-0.03	-0.21; 0.15	0.754	-0.05	-0.11; 0.02	0.158	-0.07	-0.23; 0.08	0.339	-0.02	-0.08; 0.05	0.616
**Post-COVID-19 symptoms**												
Neurological symptoms	-	-	-	0.07	0.06; 0.08	<0.001	0.25	0.22; 0.28	<0.001	0.12	0.10; 0.13	< 0.001
Digestive symptoms	0.40	0.35; 0.46	<0.001	-	-	-	0.38	0.35; 0.41	<0.001	0.20	0.18; 0.22	< 0.001
Respiratory and heart symptoms	0.43	0.41; 0.46	<0.001	0.12	0.10; 0.13	<0.001	-	-	-	0.07	0.06; 0.08	< 0.001
Other symptoms	0.55	0.51; 0.59	<0.001	0.16	0.14; 0.18	<0.001	0.18	0.17; 0.20	<0.001	-	-	-
