# Supplementary material for: Severity and geographical disparities of post-COVID-19 symptoms among the Vietnamese general population: a national evaluation
Source: Sci Rep. 2023 Mar 17;13:4460. doi: 10.1038/s41598-023-30790-x (PMC10022561; doi:10.1038/s41598-023-30790-x)
Supplement: Supplementary file 5 — Supplementary Information 5. [file 41598_2023_30790_MOESM5_ESM.pdf]

Appendix 5. Type of variables

	Variable	Definition/Indicator	Type of variable
**Outcome variable**			
Post-COVID-19 symptoms	Rash	Rash	Binary variable
	Diarrhea	Diarrhea	Binary variable
	Change in smell or taste	Change in smell or taste	Binary variable
	Abdominal pain	Abdominal pain	Binary variable
	Chest pain	Chest pain	Binary variable
	Fast beating	Fast beating	Binary variable
	Anxiety	Anxiety	Binary variable
	Changes in menstrual cycles	Changes in menstrual cycles	Binary variable
	Myalgia	Myalgia	Binary variable
	Somnipathy	Somnipathy	Binary variable
	Dyspnea	Dyspnea	Binary variable
	Cough	Cough	Binary variable
	Difficulty thinking or concentrating	Difficulty thinking or concentrating	Binary variable
	Headache	Headache	Binary variable
	Fatigue	Fatigue	Binary variable
	Post-COVID-19 symptoms	Asymptomatic, 1 symptom, 2 symptoms, 3 symptoms, and 4 or more than 4 symptoms	Ordinal variable
	Number of post-COVID-19 symptoms	Number of post-COVID-19 symptoms (0-15)	Continuous variable
	Neurological symptoms	Yes: Present at least one of 5 symptoms (fatigue, difficulty thinking or concentrating, headache, somnipathy, and anxiety).<br>No	Binary variable
	Number of neurological symptoms	Number of neurological symptoms (0-5)	Continuous variable
	Digestive symptoms	Yes: Present at least one of 3 symptoms (change in smell or taste, diarrhea, and abdominal pain).<br>No	Binary variable
	Number of digestive symptoms	Number of digestive symptoms (0-3)	Continuous variable
	Respiratory and heart symptoms	Yes: Present at least one of 4 symptoms (yspnea, cough, chest pain, fast beating).<br>No	Binary variable
	Number of respiratory and heart symptoms	Number of respiratory and heart symptoms (0-4)	Continuous variable
	Other symptoms	Yes: Present at least one of 3 symptoms (rash, myalgia, and changes in menstrual cycles).<br>No	Binary variable

	Number of other symptoms	Number of other symptoms (0-3)	Continuous variable
	Severity of COVID-19 at the onset	Asymptomatic, mild, moderate, and severe	Ordinal variable
Covariates			
Individual characteristics	Age	Age	Discrete variable
	Gender	Male/Female	Binary variable
	Provinces	Southeast region, Northern Midlands and Mountains, Red River Delta, North Central Region, South Central Coast, Central Highlands, and Mekong Delta regions	Nominal variable
	BMI index	BMI index	Continuous variable
**Behavior**	Smoking	Yes/No	Binary variable
	Alcohol usage	Yes/No	Binary variable
	Exercise after recovering from COVID-19	Yes/No	Binary variable
**Comorbidity**	Diabetes	Yes/No	Binary variable
	Cardiovascular disease	Yes/No	Binary variable
	Chronic lung disease	Yes/No	Binary variable
	Neurological disease	Yes/No	Binary variable
	Cancer	Yes/No	Binary variable
	Comorbidity	Yes: Present at least one disease mentioned above<br>No	Binary variable
Characteristics of COVID-19 infection	Time since COVID-19 onset	1 month, 1-4 months, 4-6 months, and above 6 months	Ordinal variable
	COVID-19 infection period	less than 7 days, 7-14 days, and more than 14 days	Ordinal variable
	Prevalence of COVID-19 cases per 100,000 population in a region	Prevalence of COVID-19 cases per 100,000 population in a region	Continuous variable
	Case fatality rate	Case fatality rate	Continuous variable
	Preference of post-COVID-19 care	medical examination at the hospital, medical examination, and consultation via phone App, getting health care materials at home, direct medical examination at free examination programs, calling the doctor and consulting by phone.	Nominal variable
