# Supplementary figures and images for: Severity and geographical disparities of post-COVID-19 symptoms among the Vietnamese general population: a national evaluation
Source: Sci Rep. 2023 Mar 17;13:4460. doi: 10.1038/s41598-023-30790-x (PMC10022561; doi:10.1038/s41598-023-30790-x)

Appendix 6. Map of 63 provinces in Vietnam (Cited by OnTheWorldMap)

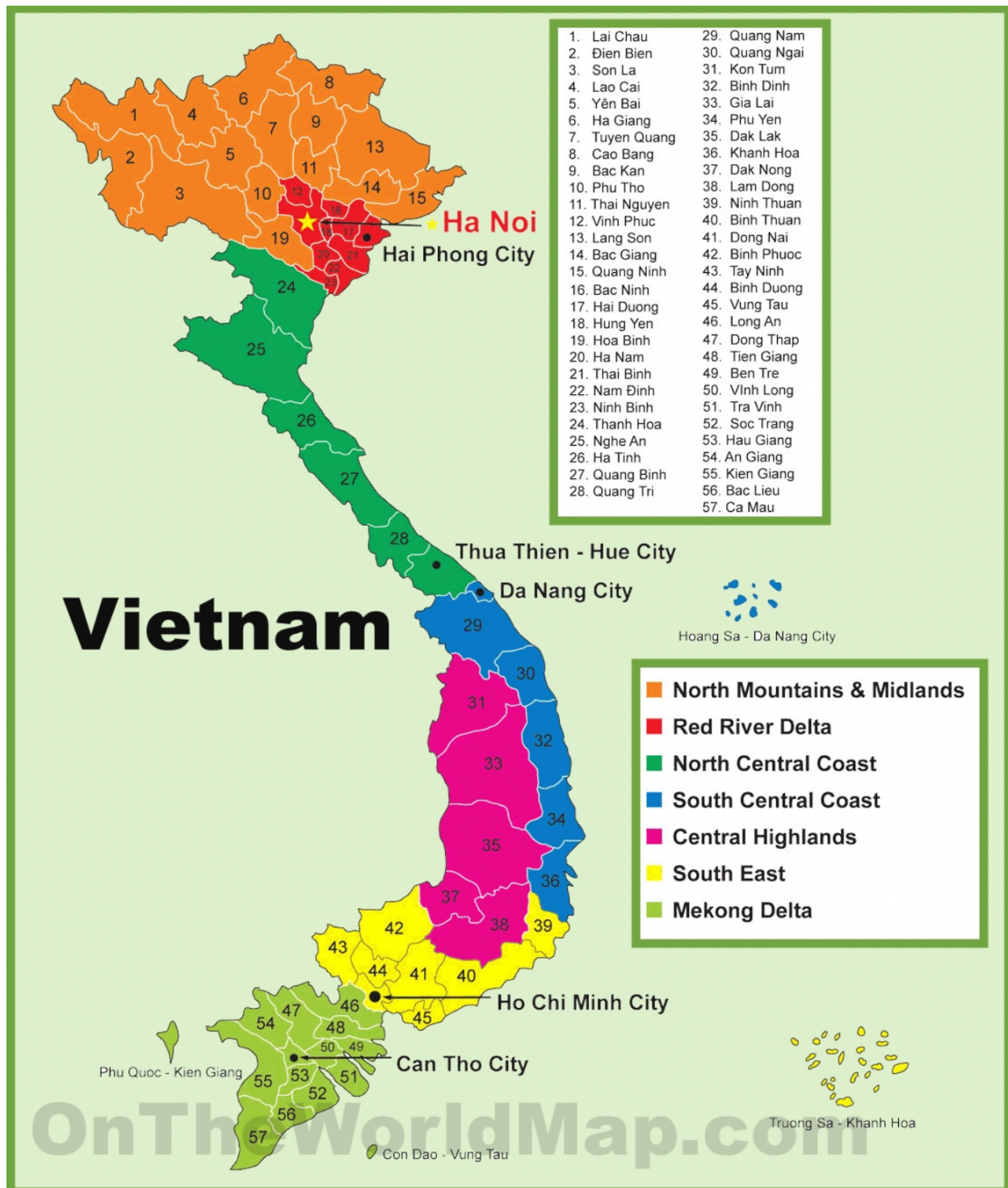

Supplement: Supplementary file 6 — Supplementary Information 6. [file 41598_2023_30790_MOESM6_ESM.pdf]
